# Surviving through solitude: A prospective national study of the impact of the early COVID-19 pandemic and a visiting ban on loneliness among nursing home residents in Sweden

**DOI:** 10.1093/geronb/gbac126

**Published:** 2022-09-02

**Authors:** Per E Gustafsson, Julia Schröders, Ingeborg Nilsson, Miguel San Sebastián

**Affiliations:** Department of Epidemiology and Global Health, Umeå University, Sweden; Department of Epidemiology and Global Health, Umeå University, Sweden; Department of Sociology, Umeå University, Sweden; Centre for Demographic and Aging Research (CEDAR), Umeå University, Sweden; Department of Community Medicine and Rehabilitation, Unit of Occupational Therapy, Umeå University, Sweden; Department of Epidemiology and Global Health, Umeå University, Sweden

**Keywords:** Interrupted time series analysis, Social relationships, Long-term care facilities

## Abstract

**Objectives:**

Targeted social distancing measures were widely implemented for nursing home residents when the extremely high COVID-19 mortality in this setting became apparent. Still, there is still scarce rigorous research examining how the pandemic and accompanying social distancing measures impacted loneliness in this group. This prospective nation-wide Swedish study of nursing home residents aimed to examine the impact on loneliness of the early phase of the pandemic and of a national visiting ban at nursing homes.

**Methods:**

A panel was selected from a total population survey of all nursing home residents in Sweden March-May 2019 and 2020 (N=11,782; age range 70-110 years; mean age 88.2 years; 71% women). Prospective pretest-posttest and controlled interrupted time series designs were employed, with time trends estimated by date of returned questionnaire. Generalized linear models were used for estimation of effects, adjusting for demographic, survey-, and health-related covariates.

**Results:**

Loneliness prevalence increased from 17 to 19% from 2019 to 2020 (Risk Ratio, RR (95% confidence interval, CI)=1.104 (1.060; 1.150)), but which was explained by self-reported health (RR(95%CI)=1.023 (0.982; 1.066)). No additional impact of the visiting ban on loneliness trends was found in the interrupted time series analyses (RR(95%CI)=0.984 (0.961; 1.008)).

**Discussion:**

The moderate but health-dependent increased risk of loneliness, and the lack of impact of the nation-wide visiting ban at nursing homes, suggest that this ostensibly vulnerable group of nursing home residents also show signs of resilience, at least during the early phase of the pandemic.

### Introduction

The emergence of the pandemic has brought urgency to the broader public health issue of loneliness among older adults (MacLeod et al., 2021), who in most countries were targeted by more restrictive social distancing measures than the population at large (Dahlberg, 2021; Dyer, 2020). This precarious situation was particularly pronounced for older adults in nursing homes, a setting that has received considerable media attention during the pandemic (Miller et al., 2021).

Nursing home residents, comprising the oldest and frailest older adults, have more limited possibilities for social interaction than other older adults due to complex health needs, functional impairments and institutional restrictions to daily life (Bethell et al., 2021; Mo & Shi, 2020; Simard & Volicer, 2020), as well as the double burden of social and digital exclusion (Seifert et al., 2021). Loneliness, a discrepancy between one's desired and achieved levels of social relations (Perlman & Peplau, 1981), is more frequent among nursing home residents than community-dwelling older adults (Gardiner et al., 2020), with prevalence estimates of severe loneliness ranging between 9 to 22% in the Nordic countries (Gardiner et al., 2020), which little variation over time (Jansson et al., 2020).

After markedly high COVID-19 mortality rate and poor survival became apparent among nursing home residents in 2020 (Ioannidis et al., 2021; Zhang et al., 2021), this group of older adults was left to weather the pandemic under particularly strict social distancing conditions, while nursing home staff battled to maintain care under the chaotic and exhausting working conditions during the first wave of the pandemic (Giri et al., 2021; Palacios-Cena et al., 2021). Despite these harsh conditions for the oldest and frailest older adults, there is a paucity of rigorous research detailing the actual impact of the pandemic and related policies on loneliness in the nursing home setting (Dahlberg, 2021; Parlapani et al., 2021). One cross-sectional study reports a marked reduction in in-person contacts during the pandemic among nursing home residents, compared to community-dwelling older adults (Freedman et al., 2021), but to our knowledge, there are no longitudinal studies on the impact on loneliness. The majority of studies on older adults include younger and community-dwelling older adults (Lebrasseur et al., 2021; Parlapani et al., 2021), with few studies on the oldest old (Simard & Volicer, 2020). Longitudinal studies on community-dwelling older adults in Europe and the USA have however mostly found weak and inconsistent impact of the pandemic on loneliness (Lebrasseur et al., 2021; Parlapani et al., 2021) and during lockdown (Fuller & Huseth-Zosel, 2021; Kotwal et al., 2021; Lin et al., 2022; Stolz et al., 2021), including in Sweden (Gustafsson et al., 2022; Kivi et al., 2021).

In Sweden, the context of the present study, the first COVID-19 pandemic wave emerged in mid-March 2020 and ended in the summer the same year. In contrast to many other countries, Sweden lacked legal possibilities to enforce compulsory and indiscriminate lockdown measures, and instead opted for a controversial strategy (Habib, 2020; Vogel, 2020) that heavily relied on voluntary recommendations of social distancing (Ludvigsson, 2020). Policies specifically targeting older adults were more restrictive, however, with strict but ultimately voluntary recommendations of quarantine for adults 70 years or older introduced promptly in mid-March 2020. After a series of devastating COVID-19 outbreaks emerged among older adults in nursing homes in Sweden, similar to the experience in other countries (Ioannidis et al., 2021), the Swedish government issued an ordinance prohibiting all external visits at nursing homes for older people.

This visiting ban, in effect from April 1st, 2020, aimed at protecting the most vulnerable group from severe and fatal consequences of COVID-19. This strategy ultimately proved unsuccessful, as the unexpectedly high COVID-19 mortality rates in Sweden compared to neighboring Nordic countries (Nanda & Sharma, 2021) have been attributed largely to the failure to protect the oldest old in nursing home facilities (Diderichsen, 2021;

Habib, 2020; The Swedish Corona Commission, 2020). Nevertheless, the visiting ban did mean that all Swedish older adults living in nursing homes overnight became deprived of physically meeting their close ones, and were left to the social contacts with staff and the other residents under the new, highly restrictive, pandemic eldercare routines (Public Health Agency of Sweden, 2020).

While the necessity of the visiting ban has not been widely questioned in Sweden, it was a nationally unprecedented measure which received criticism, e.g., for the selective restriction of autonomy and the lack of accompanying plans to counteract social isolation among nursing home residents (Dahlberg, 2021; Skoog, 2020). This critique ties into the international discussion of ageism during the COVID-19 pandemic (Ayalon et al., 2021), and the Swedish visiting ban can be seen as an example of the problematic yet necessary decisions based on a conflicting continuum of risk and harm, which have marked the societal responses to the COVID-19 pandemic (Smith et al., 2020).

The present study seeks to examine the impact of the emergence of the pandemic itself and of the visiting ban on loneliness among older adults residing in nursing homes in Sweden, using both pretest-posttest and controlled interrupted time-series (ITS) designs. The controlled ITS is considered the strongest study design to evaluate the causal effect of temporally well-defined policies in cases where randomization is prohibitive (Kontopantelis et al., 2015), and has been recommended specifically for impact evaluations of COVID-19 policies (Haber et al., 2021). The study aimed to test the following two hypotheses:

1) The emergence of the COVID-19 pandemic was associated with an increased risk of loneliness among older adults in nursing homes; and

2) The introduction of the national visiting ban at nursing homes caused an additional increased risk of loneliness, beyond the impact of the pandemic itself.

## Methods

### The Swedish nursing home setting

Nursing homes in Sweden are the responsibility of the 290 municipalities, but they can be operated by either public or private providers. A subsidized nursing home place can be granted based on assessment of individual needs, as regulated by the Social Services Act (1980:620). Places are reserved for individuals for whom the care needs are greater than those that can be provided by home care services, the first level of municipally managed and subsidized eldercare. The care professions at nursing homes varies but usually have a registered nurse with overall responsibility for care provision, with care delivered by auxiliary nurses and nurse's assistants, and with other professions such as occupational therapists, physiotherapists, and medical doctors available for consulting or visits. The total population of Swedish nursing home residents are identifiable in the Social Services Registers of The National Board of Health and Welfare.

### Study population and data collection

The data of the present study came from two waves of the Elderly Survey in Nursing Homes 2019 and 2020, which were implemented by the National Board of Health and Welfare to monitor the quality of eldercare in Sweden. The target population comprised the total population of nursing home residents aged 65 years or older in Sweden each year, in 2019 and 2020, respectively. The survey questionnaire comprises 20-25 items covering perceptions of the eldercare as well as self-rated loneliness and health. Additional demographic information, e.g., sex, provider, and municipality of residence, were linked to the survey data from total population registers.

Information about the survey, invitation to participate and the questionnaire were distributed by mail to the entire target population on March 5$^{th}$, 2020, with first responses returned on March 9$^{th}$, 2020; the same week the first signs of COVID-19 community transmission in Sweden were detected, and 23 days before the visiting ban was in effect from April 1st. Two reminders were sent out during the study period, and the last response accepted was on May 31st. The respondents had the options of completing the questionnaire in paper form and returning it by a self-addressed stamped envelope, or by the web following a web link provided in the information. Respondents could complete the questionnaire on their own, or with assistance, e.g., from nursing home staff, relative or a friend. The 2019 survey took place over the equivalent period and using the same procedures.

For the present study, all respondents who completed both the 2019 and 2020 surveys and who were 70 years or older in 2020 were included, to avoid potential bias due to the recommendations of social distancing for older adults ≥70 years. Records were kept of the date each questionnaire was sent out and returned. Questionnaires were distributed to N=72,431 nursing home residents in 2019, with N=36,248 (50%) completing the questionnaire, of whom N=35,853 were 70 years or older. Of this eligible and available population at baseline, N=15,159 (42%) also completed the questionnaire in 2020. Due to missing data on individual variables, N=11,782 individuals and N=23,564 observations were available for analysis in the longitudinal analysis (33% of the eligible and available population, and 16% of the target population at baseline). Descriptive characteristics of the analytical sample is shown in Table 1.

Ethical approval was granted by the Swedish Ethical Review Authority [Ref. No. 2020-06879].

**Study design**

For the first hypothesis, a pretest-posttest design was used by the longitudinal comparison of loneliness by *year* (1=pandemic intervention year of 2020, 0= pre-pandemic control year of 2019), i.e., a within-subject comparison of dependent observations.

For the second hypothesis, three design variables were operationalized in a controlled interrupted time series (ITS) design. The controlled ITS design is a quasi-experimental design which, in contrast to more common quasi-experimental designs relies on comparison of time trends before and after an intervention instead of single-point measures, and is therefore able to control by design biases rooted in underlying trends (Kontopantelis et al., 2015).

First, the condition of *year* was operationalized as above. Second, the return dates of the questionnaires were used to classify responses, from the 2020 intervention year as well as the 2019 control year, into two *periods;* before (0=pre-intervention period; 3 weeks up to March 31$^{st}$) and after (1=post-intervention period, 9 weeks from April 1$^{st}$) the introduction of the visiting ban in 2020. Third, *time* corresponded to the return date of questionnaire, by two-day intervals on a continuous scale. Three days adjustment was applied for physical questionnaires to take the time delay of postal service into account. As the study population comprised independent observations within each year, *period* and *time* comprised between-subject comparisons.

The ITS design relies on estimation of time trend (*time*) in risk of loneliness in the only group exposed to the visiting ban (intervention *period*, during the 2020 intervention *year*). To estimate the intervention impact, this trend is first compared to the corresponding trend in the group responding before the introduction of the visiting ban (pre-intervention *period*, during the same 2020 intervention *year*). To take unmeasured confounding into account, the resulting pre-post trend difference is subsequently compared to the corresponding pre-post trend difference the preceding year (pre-intervention and intervention *period*, during the 2019 control *year*), which due to the longitudinal design represents a within-subject comparison. The resulting estimate of the intervention impact thus amounts to a difference-in-difference of trends in the risk of loneliness. The design is schematically illustrated in Figure 1.

### Measures

*Perceived loneliness*

*Loneliness*, the outcome of interest, was measured by a single questionnaire item 'Does it happen that you are troubled by loneliness?', which was constructed for this survey. It has Likert-scale response options which were dichotomized (1= 'Yes, often'; 0='Yes, now and then' or 'No'). The 'Don't know/No opinion' responses were coded as '0' if the questionnaire completed by respondent and as missing if completed with assistance of another person. The rationale was that individuals themselves selecting this option are unlikely to suffer from frequent loneliness, but if completed with help of another person it may also reflect unwillingness to disclose loneliness or communication difficulties.

*Covariates*

Demographic and survey-related characteristics that potentially could be related to the design conditions and simultaneous affect perceived loneliness were measured both in 2019 and 2020 and included as covariates: nursing home *provider* (0=public/1=private); *questionnaire form* (0=web, 1=physical) and *questionnaire assistance*, i.e., whether the respondent completed the questionnaire independently or with assistance (0=no/1=with assistance); *area of residence* (0=large city, 1=medium-sized town, 2=smaller town or rural area (Swedish Association of Local Authorities and Regions, 2016)); *age* (continuous, in years); and s*ex* (0=woman, 1=man) .

Poor health is both a potential consequence (Luo et al., 2012), and cause (Dahlberg et al., 2022) of loneliness, and its status as confounder is therefore ambiguous. For this reason, the following health measures were included as covariates only in separate models: *self-rated health*, based on five Likert-scale response option dichotomized into 0='Fair', 'Quite good' and 'Very good' vs 1='Quite bad' and 'Very bad'; *Worries and anxiety problems*, based on three Likert-scale response options dichotomized into 0='No' vs 1= 'Mild' or 'Severe troubles'; and indoor physical *mobility limitations*, based on four Likert response options dichotomized into 0='No limitations' or 'Certain limitations' vs 1='Considerable difficulties' or 'No mobility'.

**Statistical analysis**

All analyses were run on the longitudinal sample of N=23,564 observations (11,782 individuals), using generalized linear model, with binomial family and log link for estimation of risk ratios (RR), in Stata. Dependency of observations between 2019 and 2020 owing to the longitudinal sample was handled by estimation of cluster-robust standard errors.

The first hypothesis was tested by regressing loneliness on *year* (1=2020 intervention; 0= 2019 control) and covariates in both years, in the longitudinal sample. Analyses were run in three models: separate crude/unadjusted models for each independent variable (Model 0), one model adjusting for demographic and survey-related covariates (Model 1; excluding sex and age), and lastly one model adding health measures (Model 2). Due to the longitudinal sample, sex was the same both years age differed by a one-year constant for all individuals. Sex and age could therefore not confound the main effect of *year* and were therefore not included in the adjusted analyses.

For the second hypothesis, interaction terms were first constructed from the main effect variables *year* (1=2020 intervention; 0= 2019 control), *period* (0=pretest; 1=posttest), *time* (continuous, 2-day intervals of date of returned questionnaire), representing all two-way (*year* × *period*; *year* × *time*; *period* × *time*) and the three-way (*year* × *period* × *time*) interactions, as described by Linden (2015). The binary outcome *loneliness* was then regressed on all main and interaction terms. The *year* × *period* × *time* interaction term is the parameter of main interest for the hypothesis, as it corresponds to the difference-in-difference in loneliness trends between the post-intervention period in 2020 and the pre-intervention period and corresponding periods in 2019. Three models were run: crude/unadjusted model

(Model 0), adjusting for demographic and survey-related covariates (Model 1; including sex and age), and lastly adding health measures (Model 2). Sensitivity analyses were conducted specifying the impact of the visiting ban on loneliness two weeks after its introduction, on April 15$^{th}$ instead of April 1$^{st}$.

Dropout analysis (see Supplementary Table S1) on the eligible study population in 2019 (N=35,853) indicated that the risk of missingness in 2020 was strongly predicted by baseline high age (RR (95%CI) = 1.208 (1.155; 1.265) for 100 years or older), poor general health (RR (95%CI) = 1.123 (1.103; 1.144)), mobility limitations (RR (95%CI) = 1.144 (1.124; 1.165)) and questionnaire assistance (RR (95%CI) = 1.088 (1.068; 1.109)). All other baseline characteristics were RR<1.04.

### Results

Descriptive statistics of the study population for 2019 and 2020 are displayed in Table 1. The sample comprised 71% women and had a mean age of 88.2 years in 2020. There were minimal changes in residential circumstances between the years, but a higher proportion completing the questionnaire with aid and by the web in 2020 compared to 2019. Mental health symptoms were markedly frequent already in 2019 (61%), and, together with general health and mobility limitations, deteriorated from 2019 to 2020.

Corresponding to the first hypothesis, that the emergence of the pandemic was associated with an increased risk of loneliness, Table 2 shows a summary of analyses with loneliness regressed on year (2020 vs. 2019) and covariates. The crude model (Model 0) showed a significant risk ratio (RR)=1.104 (95%CI: 1.060; 1.150) for the *year* effect, which corresponds to the small descriptive increase in loneliness prevalence from 2019 (17%) to 2020 (19%), as reported in Table 1. This estimate was attenuated to RR=1.080 (95%CI: 1.036; 1.125) after adjustment for covariates (Model 1), and further to a non-significant RR=1.023 (95%CI: 0.982; 1.066) after taking variations in health into account (Model 2).

Adjusting only for one health measure at a time in separate models (see Supplementary Table S2) yielded similar inferences but with attenuation of the main *year* effect below significance only when adjusted for self-rated health (RR (95%CI) = 1.036 (0.994; 1.080)), with smaller attenuations when adjusting for either mental health (RR (95%CI) = 1.054 (1.011; 1.098)) or mobility limitations (RR (95%CI) = 1.052 (1.009; 1.097)).

Of the covariates, mental health was the strongest independent predictor (RR (95%CI) = 3.717 (3.395; 4.070) in Model 2), with general health and mobility limitations displaying weaker but significant relationships. Those responding to questionnaire with assistance and by the web reported slightly higher risk of loneliness, but no independent association was found with area of residence and provider in adjusted analyses (Model 2). While age and sex were not included as covariates in the adjusted analyses, crude analyses (Model 0) indicated that men on average reported lower risk of loneliness than women, with no effect of age.

A summary of the analyses corresponding to the second hypothesis, that the visiting ban at nursing homes led to an increased risk of loneliness, is shown in Table 3. The main parameter of interest, the *Year* × *Period* × *Time* effect, was in the crude model (Model 0) estimated at RR(95%CI) = 0.974 (0.949; 0.998), which indicated a relative decrease in loneliness trends (RR=0.974 (95%CI: 0.949; 0.998)) after the introduction of the visiting ban, compared to the period before the visiting ban and to the trends of the corresponding periods in 2019. The estimate was attenuated below significance after adjustment for covariates (Model 1), and further attenuated to a non-significant RR=0.984 (95%CI: 0.961; 1.008) after considering health in 2019 and 2020 (Model 2). The other estimates in the models indicated higher baseline prevalence of loneliness in 2020 (*Year*) but which was attenuated below significance after adjustments.

Sensitivity analyses setting the policy impact two weeks after the introduction of the visiting ban, on April 15th, led to similar inferences (see Supplementary Table S3). In these analyses, the *Year* × *Period* × *Time* effect was non-significant throughout the models.

### Discussion

In the present national longitudinal study of Swedish older adults residing in nursing homes in both 2019 and 2020, we found support for our first hypothesis of increased risk of loneliness during the Covid-19 pandemic, which however was explained by a simultaneous deterioration in health from 2019 to 2020. We found no support for our second hypothesis of increased risk of loneliness specifically attributable to the implementation of the nation-wide visiting ban at nursing homes. Taken together, the results point to a moderate and health-dependent immediate impact on loneliness of the pandemic and no impact of a restrictive policy, in this group of older adults ostensibly vulnerable to the psychosocial consequences of the pandemic.

We found markedly high frequency of worries and anxiety already before the pandemic, which corresponds to prior findings of high prevalence of depressive symptoms among nursing home residents (Baxter et al., 2022). Mental health, along with self-rated health and mobility limitations, deteriorated from 2019 to 2020, and the increased risk of loneliness in 2020 was largely dependent on this concurrent deterioration in health. This pattern may reflect the bidirectional relationship of loneliness and self-perceived health among older adults in the nursing home setting (Bethell et al., 2021; Lapane et al., 2022), and among older adults in general (Dahlberg et al., 2022). This finding can therefore both illustrate that preserving good health and functioning can be crucial for preventing experiences of loneliness, and that social interactions are important for well-being, for nursing home residents during the pandemic.

The prevalence of loneliness in both 2019 (17%) and 2020 (19%) is comparable to estimates in the home care setting in the Nordic countries before the pandemic (Gardiner et al., 2020). However, as the prevalence of loneliness in the nursing home setting tends to be stable across years (Jansson et al., 2020), the increased risk we found, from one year to another, remains concerning. At the same time, considering the tumultuous conditions at nursing homes during the early phase of the pandemic (Ioannidis et al., 2021; Xu et al., 2020), the magnitude of increased risk found can be seen as surprisingly moderate, particularly as we found no evidence of any additional increased risk of loneliness following the introduction of the national visiting ban.

While previous research has found a notable reduction in in-person contacts for nursing home residents during the pandemic (Freedman et al., 2021), research on loneliness is dominated by studies of community-dwelling older adults. Interestingly, this body of literature has also, overall, found a comparably modest or lack of impact of the pandemic on loneliness (Lebrasseur et al., 2021; Parlapani et al., 2021), including from Sweden (Gustafsson et al., 2022; Kivi et al., 2021), with one Swedish study also failing to find any impact of social distancing recommendations on loneliness among older adults with home care (Gustafsson et al., 2022). The present study gives credence to the voices challenging the view of older adults, and particularly nursing home residents, being singularly vulnerable to the psychosocial impact of the pandemic and related policy measures (Losada-Baltar et al., 2021; Parlapani et al., 2021). However, there are several potential explanations to the finding of a moderate impact of the pandemic conditions, with methodological causes discussed further below.

First, nursing home residents may engage in more social interaction than their community-dwelling counterparts due to the daily interactions with nursing home staff (Adlbrecht et al., 2021), which have been shown to be vital for the residents' well-being

(Kang et al., 2020; Liu et al., 2020). This feature may have protected many residents against experiences of loneliness during the early phase of the pandemic, despite the physical absence of family and the relative social isolation that the visiting ban enforced.

Second, despite the risk of digital exclusion among nursing home residents (Seifert et al., 2021), it is plausible that families made additional efforts to maintain social interactions with their family members at nursing homes during the pandemic, e.g., increasing frequency of phone or video calls (Giri et al., 2021). The Swedish visiting ban did gain considerable public attention as it was, in the Swedish context, an unprecedented measure that was quite distinct from the more lenient general pandemic strategy, and which indirectly affected the entire population.

Third, it is possible that many nursing home residents and their families had limited their social interactions already ahead of the introduction of the visiting ban. Public awareness of the COVID-19 pandemic was high even before community spread of the virus was first observed in Sweden in mid-March 2020, and the promptly disseminated recommendations to older adults were generally adhered to (Gustavsson & Beckman, 2020). This possibility is supported by the higher baseline loneliness in 2020 than in 2019 found in this study.

Fourth, the present study only covered the first three months of the COVID-19 pandemic, and the experience of loneliness could take longer time to develop. While sensitivity analyses of the visiting ban led to similar inferences, other studies of community-dwelling older adults have found loneliness increasing not until later during pandemic (Hansen et al., 2021).

**Methodological considerations**

The strengths of the study include the large sample from a total population survey; the inclusion of the understudied target population of the oldest old in nursing homes; the prospective design and within-subject comparisons enabling control of a range of potential confounders by design, e.g., traits, persistent social conditions, and seasonal variations; and the rigorous controlled ITS design which gives strong support for causal inference. Nevertheless, research on populations of the oldest old (Dahlberg, 2021), and particularly evaluative research during the pandemic (Haber et al., 2021), faces multiple challenges which also apply to the present study.

First, the target population was older adults in nursing homes, and results should not be generalized to the wider population of older adults. As for all comparable studies conducted, the heterogenous arrangements of eldercare and societal responses to the COVID-19 pandemic across countries also limit generalizability to other societal contexts.

Second, while the survey was disseminated to the entire target population of nursing home residents in Sweden, only 50% responded to the survey at baseline, of whom only one third was retained for longitudinal analysis. Selection due to frailty, cognitive and communication problems is a common challenge in studies of the oldest old (Dahlberg, 2021), and the high mortality in nursing homes during the first wave of the pandemic risks introducing survival bias. Even though information on these characteristics were not available in this study, drop-out analysis confirmed a systematic attrition by baseline age, health and functioning. This bias would be expected to lead to an underestimation of the impact of the pandemic and the visiting ban, and limits generalizability to nursing home residents in general.

Third, the ITS design is susceptible to misspecified timing of the policy impact (Haber et al., 2021). While the studied intervention was introduced as a national law on a specific date in all nursing homes, there is uncertainty as to which degree and when any objective social isolation was perceived as loneliness among the nursing home residents. While sensitivity analyses specifying the policy impact to two weeks after its introduction corroborated the inferences, a possible lag in the population impact remains an additional uncontrolled potential source of bias that is most likely expressed as an underestimation of the impact of the visiting ban.

Fourth, the ITS design relies on the counterfactual assumptions of projected and parallel trends (Haber et al., 2021) which are difficult to assume especially in the light of the extraordinarily chaotic conditions of the first wave of the COVID-19 pandemic, where also other competing interventions, or changes cooccurring with the studied intervention, could introduce bias (Haber et al., 2021). Although the competing impact of the recommendations to all older adults disseminated two weeks before the visiting ban in theory is methodologically managed by consideration of the pre-intervention trends, the possibility of lags in impact, as discussed above, makes it impossible to rule out it as a competing intervention and a source of bias.

Fifth, the study used a single-item measure of loneliness, which has not been subjected to psychometric evaluations and its validity and reliability can therefore not be ascertained. We adjusted for assistance with questionnaire completion to take into account potential response bias. The time frame of loneliness is not specified in the questionnaire, which would be expected to introduce random and possibly systematic error to the measurement. Moreover, unmeasured confounders, such as length of time in the nursing home, could potentially bias the findings.

**Conclusions and policy implications**

Taken together, the present prospective national study suggests that Swedish older adults residing in nursing homes in 2019 and through the initial months of the COVID-19 pandemic in 2020, did not experience increased prevalence of loneliness following the national visiting ban at nursing homes, despite the sharp restrictions to external social contacts it entailed. A noticeable increase in the risk of loneliness compared to the year prior was largely explained by a simultaneous deterioration in health, which can reflect that the first pandemic wave still affected nursing home residents with poor health. There is still a marked scarcity of rigorous studies on how the oldest and frailest older adults, including nursing home residents, have fared psychosocially during the COVID-19 pandemic, particularly when it comes to the impact of policies. To provide clearer guidance for public health and eldercare policy and practice, more research is needed to evaluate particularly the long-term impact of the sustained pandemic and social distancing measures in this group.

**Table 1.** Descriptive statistics the longitudinal sample of Swedish nursing home residents. Numbers are N (%) individuals if not otherwise noted.

| Variable | Pre-pandemic survey (2019) | Pandemic survey (2020) |
|---|---|---|
| Total sample, individuals (observations) | 11,782 (23,564) | |
| Pre-intervention period | 9,009 (76) | 7,933 (67) |
| Post-intervention period | 2,773 (24) | 3,849 (33) |
| Gender | | |
| Women | 8,319 (71) | |
| Men | 3,464 (29) | |
| Age (years) in 2020 | | |
| M (SD) | 88.2 (7.3) | |
| Range | 70 - 110 | |
| Area of residence | | |
| Large City | 3,244 (28) | 3,251 (28) |
| Medium-sized town | 4,820 (41) | 4,821 (41) |
| Small town or rural | 3,718 (32) | 3,710 (31) |
| Provider | | |
| Private | 2,457 (21) | 2,417 (21) |
| Public | 9,325 (79) | 9,365 (79) |
| Questionnaire form | | |
| Postal | 10,928 (93) | 10,200 (87) |
| Web | 854 (7.3) | 1,582 (13) |
| Questionnaire assistance | | |
| No | 5,239 (44) | 4,771 (40) |

| | | |
|---|---|---|
| Yes | 6,543 (56) | 7,011 (60) |
| Self-rated health | | |
| Good | 8,768 (74) | 8,283 (70) |
| Poor | 3,014 (26) | 3,499 (30) |
| Mental health symptoms | | |
| No | 4,653 (39) | 4,248 (36) |
| Yes | 7,129 (61) | 7,534 (64) |
| Mobility limitations | | |
| No | 7,165 (61) | 6,332 (54) |
| Yes | 4,617 (39) | 5,450 (46) |
| Loneliness | | |
| Never or sometimes | 9,755 (83) | 9,544 (81) |
| Yes, often | 2,027 (17) | 2,238 (19) |

**Table 2.** Relative risk of loneliness from 2019 to 2020 among Swedish nursing home residents. Numbers are risk ratios with 95% confidence interval.

| Variable | Model 0[a] | Model 1[b] | Model 2[c] |
|---|---|---|---|
| Year | | | |
| 2020 (ref: 2019) | 1.104 (1.060; 1.150) | 1.080 (1.036; 1.125) | 1.023 (0.982; 1.066) |
| Area (ref: large city) | | | |
| Medium-sized town | 0.953 (0.881; 1.031) | 0.966 (0.891; 1.046) | 1.000 (0.929; 1.076) |
| Small town or rural | 0.928 (0.853; 1.010) | 0.938 (0.86; 1.022) | 0.973 (0.898; 1.054) |
| Provider | | | |
| Public (ref: private) | 0.943 (0.872; 1.019) | 0.962 (0.888; 1.042) | 0.953 (0.885; 1.026) |
| Questionnaire form | | | |
| Web (ref: postal) | 1.210 (1.108; 1.320) | 1.153 (1.055; 1.26) | 1.095 (1.009; 1.189) |
| Questionnaire assistance | | | |
| Yes (Ref: no) | 1.385 (1.300; 1.476) | 1.374 (1.289; 1.465) | 1.063 (1.009; 1.130) |
| Self-rated health | | | |
| Poor (ref: good) | 2.483 (2.343; 2.631) | – | 1.788 (1.68; 1.903) |
| Mental health symptoms | | | |
| Yes (ref: no) | 4.489 (4.075; 4.946) | – | 3.713 (3.363; 4.099) |
| Mobility limitations | | | |
| Yes (ref: no) | 1.571 (1.477; 1.670) | – | 1.132 (1.063; 1.207) |
| Sex | | | |
| Man (ref: woman) | 0.885 (0.822; 0.952) | – | – |
| Age (per year of age) | 0.988 (0.994; 1.002) | – | – |

*Note.* N=23,564 observations

[a] Model 0: nine unadjusted models separate for each independent variable;

[b] Model 1: adjusted for living alone, area of residence, provider, questionnaire form, and questionnaire assistance;

[c] Model 2: additionally adjusted for self-rated general health, mental health and mobility limitations

**Table 3.** Results of controlled interrupted time series analyses of the impact of a visiting ban on loneliness among Swedish nursing home residents. Numbers are risk ratios with 95% confidence intervals.

| **Effect** | **Model 0**[a] | **Model 1**[b] | **Model 2**[c] |
|---|---|---|---|
| Year (2020 vs 2019) | 1.266 (1.078; 1.486) | 1.234 (1.053; 1.446) | 1.070 (0.919; 1.245) |
| Period (post vs. pretest) | 1.096 (0.908; 1.323) | 1.048 (0.869; 1.265) | 0.968 (0.809; 1.158) |
| Time (date, 2-day intervals) | 0.994 (0.978; 1.012) | 0.995 (0.978; 1.012) | 0.998 (0.982; 1.014) |
| Year × Period | 1.236 (0.886; 1.725) | 1.223 (0.878; 1.702) | 1.251 (0.913; 1.715) |
| Year × Time | 1.029 (1.007; 1.052) | 1.027 (1.006; 1.049) | 1.016 (0.996; 1.037) |
| Period × Time | 1.009 (0.989; 1.029) | 1.010 (0.99; 1.030) | 1.010 (0.991; 1.029) |
| Year × Period × Time | 0.974 (0.949; 0.998) | 0.975 (0.951; 0.999) | 0.984 (0.961; 1.008) |

*Note.* N=23,564 observations

[a]Model 0: one unadjusted model only including the design effects;

[b]Model 1: adjusted for living alone, area of residence, provider, questionnaire form, questionnaire assistance, age and sex

[c]Model 2: additionally adjusted for self-rated general health, mental health and mobility limitations

**Figure 1.** Overview of the controlled interrupted time series design illustrating the four design conditions by year and period. Time trends in risk of loneliness are estimated within and compared between the four conditions.

Accepted Ma

## Supplementary Material

gbac126_suppl_Supplementary_Tables

