# Supplementary material for: Surviving Through Solitude: A Prospective National Study of the Impact of the Early COVID-19 Pandemic and a Visiting Ban on Loneliness Among Nursing Home Residents in Sweden
Source: J Gerontol B Psychol Sci Soc Sci. 2022 Sep 2;77(12):2286–95. doi: 10.1093/geronb/gbac126 (PMC9494329; doi:10.1093/geronb/gbac126)
Supplement: gbac126_suppl_Supplementary_Tables [file gbac126_suppl_supplementary_tables.pdf]

Table S1. Dropout analysis predicting risk of missingness in 2020 by baseline characteristics in 2019 in unadjusted analyses. Numbers are risk ratios with 95% confidence interval.

Variable	Model 0[a]
Loneliness	
Yes, often (ref: Never/sometimes)	1.037 (1.012; 1.062)
Area (ref: large city)	
Medium-sized town	0.983 (0.963; 1.005)
Small town or rural	0.983 (0.961; 1.006)
Provider	
Public (ref: private)	0.998 (0.977; 1.02)
Questionnaire form	
Web (ref: postal)	1.014 (0.982; 1.047)
Questionnaire assistance	
Yes (Ref: no)	1.088 (1.068; 1.109)
Self-rated health	
Poor (ref: good)	1.123 (1.103; 1.144)
Mental health symptoms	
Yes (ref: no)	0.992 (0.974; 1.01)
Mobility limitations	
Yes (ref: no)	1.144 (1.124; 1.165)
Sex	
Man (ref: woman)	1.035 (1.016; 1.055)
Age (ref: 70-79 years)	
80-89 years	1.038 (1.007; 1.07)
90-99 years	1.119 (1.087; 1.153)

100 years or older	1.208 (1.155; 1.265)

Note. N=32,342 - 35,853 observations

[a] Model 0: separate unadjusted models for each independent variable.

Table S2. Relative change in loneliness from 2019 to 2020 among Swedish nursing home residents by single adjustment for health measures. Numbers are risk ratios with 95% confidence interval.

Variable	Model 1[a]	Model 2[b]	Model 3[c]
Year			
2020 (ref: 2019)	1.036 (0.994; 1.080)	1.054 (1.011; 1.098)	1.052 (1.009; 1.097)
Area (ref: large city)			
Medium-sized town	0.982 (0.910; 1.060)	0.990 (0.918; 1.068)	0.979 (0.905; 1.060)
Small town or rural	0.960 (0.883; 1.043)	0.955 (0.879; 1.036)	0.954 (0.875; 1.039)
Provider			
Public (ref: private)	0.965 (0.893; 1.042)	0.952 (0.882; 1.027)	0.956 (0.883; 1.035)
Questionnaire form			
Web (ref: postal)	1.157 (1.064; 1.259)	1.075 (0.987; 1.171)	1.147 (1.051; 1.252)
Questionnaire assistance			
Yes (Ref: no)	1.192 (1.119; 1.269)	1.166 (1.096; 1.240)	1.29 (1.209; 1.375)
Health			
Poor (ref: good)	2.400 (2.262; 2.546)	4.363 (3.957; 4.811)	1.501 (1.410; 1.598)

Note. N=23,546 observations

[a] Health: Self-rated general health

[b] Health: Mental health symptoms

[c] Health: Functional limitations

Table S3. Results of controlled interrupted time series analyses of the impact of a visiting ban on loneliness among Swedish nursing home residents, with intervention impact set at April 15th . Numbers are risk ratios with 95% confidence intervals.

Effect	Model 0[a]	Model 1[b]	Model 2[c]
Year (2020 vs 2019)	1.159 (1.057; 1.27)	1.142 (1.043; 1.25)	1.084 (0.994; 1.182)
Period (post vs. pretest)	1.255 (0.937; 1.681)	1.233 (0.923; 1.649)	1.219 (0.925; 1.607)
Time (date, 2-day intervals)	1.000 (0.989; 1.011)	0.998 (0.987; 1.009)	0.996 (0.986; 1.006)
Year × Period	0.880 (0.470; 1.650)	0.865 (0.464; 1.613)	0.890 (0.490; 1.619)
Year × Time	1.017 (1.004; 1.03)	1.016 (1.003; 1.029)	1.017 (1.005; 1.03)
Period × Time	0.993 (0.973; 1.014)	0.996 (0.976; 1.017)	1.000 (0.981; 1.020)
Year × Period × Time	0.995 (0.970; 1.021)	0.995 (0.971; 1.021)	0.995 (0.971; 1.019)

Note. N=23,546 observations

[a]Model 0: one unadjusted model only including the design effects;

[b]Model 1: adjusted for living alone, area of residence, provider, questionnaire form, questionnaire assistance, age and sex

[c]Model 2: additionally adjusted for self-rated general health, mental health and mobility limitations
